# Identification of Potential Abuse in Young Children with a Hand Fracture

**DOI:** 10.1007/s40653-025-00750-w

**Published:** 2025-09-03

**Authors:** Merav Ben Natan, David Maman, Yaron Berkovich

**Affiliations:** 1 Hillel Yaffe Academic School of Nursing, P.O.B. 169, Hadera, 38100 Israel; 2 Nursing Department, Tel Aviv University, Tel Aviv, Israel; 3 The Orthopedic Department, Carmel Medical Center, Haifa, Israel; 4 The Orthopedics B Department, Hillel Yaffe Medical Center, Hadera, Israel; 5 Medical Department Technion University, Haifa, Israel

**Keywords:** Child abuse, Hand fracture, Young children, Risk factors

## Abstract

**Purpose:**

Identifying child abuse in young children, especially through hand fractures, presents a significant challenge for healthcare providers. This study aimed to explore the socio-demographic and medical characteristics associated with child abuse in children under five who presented with hand fractures at a pediatric emergency department in north-central Israel from 2018 to 2023.

**Methods:**

This retrospective study analyzed data from 828 children who presented with hand fractures. Key factors, such as nationality, history of previous fractures, and recent hospitalizations, were examined for their association with child abuse. Statistical analyses, including logistic regression, were performed to determine associations and significance.

**Results:**

The study identified 30 (3.6%) victims of abuse among the cohort. Significant factors associated with a higher likelihood of abuse included Arab nationality (OR 2.4; 95% CI 1.01–5.93), prior fracture history (OR 1.9; 95% CI 1.20–2.99), and hospitalization within the past year (OR 3.1; 95% CI 1.31–7.46), all with *p*-values < 0.01. Victims of abuse had more previous fractures (M = 0.93) and more hospitalizations in the previous year (M = 1.13) compared to non-victims. In the reviewed cases classified as abuse, documentation indicated the father as the alleged perpetrator in each instance.

**Conclusions:**

These findings highlight the critical role of healthcare providers in recognizing and reporting potential child abuse, particularly in cases of recurrent injuries and frequent hospital visits. Understanding these risk factors can help in early identification and protection of at-risk children. Further research is needed to enhance protective measures and gain deeper insights into the factors influencing the identification of child abuse.

## Introduction

Child abuse represents a significant public health issue with profound implications for both individuals and society (Özbay et al., 2024). It can lead to severe morbidity and mortality, often going unrecognized until significant injuries become apparent. Early identification of abuse is critical for protecting children and preventing fatalities and serious, irreversible outcomes (Michaels & Letson, 2021).

Physical abuse accounts for an estimated 12–20% of fractures in infants and toddlers (Marine & Forbes-Amrhein, 2021). However, nearly half of children suffering from fractures due to abuse are not initially recognized as abuse victims. Misdiagnosis can occur due to the similarities in symptoms between accidental and abusive injuries, compounded by inconsistent or vague explanations from caregivers. A lack of awareness or experience among healthcare providers in recognizing abusive trauma, along with cultural misunderstandings, can impede accurate diagnosis. Moreover, the failure to recognize patterns in previous injuries and inadequate collaboration among healthcare providers, social workers, and law enforcement often result in missed opportunities to identify abuse (Manan et al., 2022). Identifying the clinical, developmental, and historical features that differentiate abusive from non-abusive fractures, particularly in young children with humerus injuries—is essential for improving diagnostic accuracy and protecting vulnerable children (Rosado et al., 2017).

Hand fractures are common among children, with an annual incidence ranging from 26.4 to 44.8 per 10,000 children. These fractures are most prevalent in two age groups: toddlers and adolescents. In toddlers, hand injuries often result from crush incidents or entrapment, while the second peak in incidence between the ages of 13 and 15 corresponds to increased participation in sports activities (Arneitz et al., 2023).

Hand fractures can be specific indicators of abuse, particularly when there is no history of trauma to the hands (Karmazyn et al., 2011; Kleinman et al., 2013; Lindberg et al., 2013). Despite their potential as indicators of abuse, there is a lack of research on the factors associated with the identification of abusive hand fractures, especially when compared to other types of injuries.

The aim of this study is to determine the socio-demographic and medical characteristics associated with identification of child abuse in young children presenting to the emergency department (ED) with hand fractures. By analyzing these characteristics, the study may reveal healthcare providers’ practices in distinguishing between abusive and accidental fractures. This insight has the potential to lead to enhanced protocols for identifying and responding to child abuse, thereby safeguarding at-risk children more effectively.

This study offers a novel contribution by focusing on hand fractures - an underexplored injury type in the context of child abuse detection. Unlike prior work that often relies on clinical suspicion alone, our analysis is grounded in real-world data combining electronic medical records with formally substantiated abuse reports submitted to the Ministry of Health. This methodological rigor enhances diagnostic certainty. Furthermore, by examining a demographically diverse population in a unique sociopolitical setting, our findings provide culturally relevant insights into potential disparities in abuse identification, which are often overlooked in international literature.

## Methods

### Study Design and Population

This retrospective descriptive study analyzed medical records of 828 children aged five years or younger who presented with hand fractures to the pediatric ED of a 552-bed hospital in the north center of Israel, between 2018 and 2023. Inclusion criteria were children aged five years or younger who presented with hand fractures and had follow-up records.

The medical records of eligible participants were reviewed to gather comprehensive data. This included socio-demographic information such as age, gender, and nationality. Additionally, medical details regarding the cause of the fracture, results of imaging studies, and any surgical interventions were collected. The records also provided insights into the medical history, such as the number of previous fractures and hospitalizations in the year prior to the presentation. Outcomes included the length of hospital stay, instances of rehospitalization, and mortality.

Further, information on reports to social services and details about the alleged perpetrator were also recorded. When a child with a hand fracture presents to the pediatric ED, a structured protocol is followed to identify potential child abuse. This protocol involves a series of steps to ensure thorough evaluation: initial assessment, reporting, investigation, reports to social services, and substantiation.

Initial Assessment: Health professionals, including pediatricians and emergency physicians, perform a comprehensive clinical evaluation of the child. During this assessment, suspicions of abuse may arise based on several clinical criteria. These include inconsistencies in the child’s account of the injury, patterns of fractures that do not align with typical accidental injuries, or signs of neglect or maltreatment.

#### Reporting

If abuse is suspected, the case is reported to the hospital’s child protection team. This team, comprised of social workers and child protection specialists, is specifically trained to handle and investigate cases of suspected abuse.

#### Investigation

The child protection team conducts a thorough investigation, which involves interviewing the child’s caregivers, reviewing the child’s medical history, and sometimes involving law enforcement if necessary. This investigation aims to gather detailed information to determine whether abuse has occurred.

#### Reports To Social Services

Based on the investigation’s findings, reports are made to social services or child protective services in accordance with legal and institutional protocols. These reports help ensure that appropriate measures are taken to protect the child.

#### Substantiation

The final determination of whether abuse has occurred is based on the child protection team’s findings. The outcomes of these investigations are documented in the child’s medical records. The study tracks whether cases were substantiated as abuse or deemed non-abusive based on these reports. Ethical approval for the study was obtained from the institutional IRB.

### Statistical Analysis

The statistical data analysis was conducted using SPSS software. Initially, a descriptive analysis was performed, which included calculating frequencies, means, standard deviations, and percentages. Subsequently, chi-square tests and t-tests for independent samples were applied to identify factors associated with child abuse. A logistic regression analysis was conducted to examine the predictive capability of these factors, with the odds ratio (OR) providing a measure of association between the predictors and the likelihood of being identified as a victim of child abuse. A *p*-value of less than 0.05 was considered statistically significant.

## Results

The mean age of the children was 3.41 years (SD = 1.36), with the age range spanning from one to five years. Slightly more than half were male (*n* = 459, 55.4%). Slightly more than half were Jewish (*n* = 489, 59.1%). All children arrived at the ED directly from their homes. The most common cause of fractures was falls, accounting for 68.5% (*n* = 567) of the cases. All children underwent X-rays, with 1.6% (*n* = 13) requiring a CT scan. In cases where a CT scan was performed (*n* = 13), the indication was based on clinical judgment following signs suggestive of more complex injuries. Specifically, CT imaging was ordered when children presented with concerning neurological symptoms (e.g., vomiting, altered consciousness, seizures), when there was evidence of multiple injuries, or when the X-ray findings were inconclusive but clinical suspicion remained high. These criteria are consistent with guidelines from pediatric radiology and child protection literature, which recommend CT imaging in the presence of suspected head trauma or when skeletal injuries are suspected but not clearly visualized on standard radiographs (Haney et al., 2025).

None of the participants underwent a skeletal survey or a bone scan. Surgery was needed for 6.9% (*n* = 57) of the children. A small number of children were hospitalized (*n* = 21, 2.5%). Among them, approximately half (*n* = 11, 52%) stayed for two days. Among the children who required hospitalization (*n* = 21), the majority had additional clinical complexities beyond isolated hand fractures. In several cases, surgical intervention was necessary due to multisystem trauma or co-occurring injuries. These cases often involved more extensive evaluation and multidisciplinary management. 4% of the children (*n* = 33) required rehospitalization within the year following their fracture. Notably, there were no fatalities in this cohort.

A minority of children had a history of previous fractures (*n* = 50, 6%). Additionally, a small fraction (*n* = 62, 7.5%) had been hospitalized in the year leading up to their current fracture. Thirty children, or 3.6%, had documented instances of domestic violence in their medical records, all of which were reported to social services. In our study, the classification of a child as a victim of abuse was based on an official designation recorded in Israel’s national medical records system, following a multidisciplinary evaluation conducted by a hospital social worker and, when relevant, in consultation with child protection authorities. These determinations typically rely on a combination of clinical findings, caregiver explanations, psychosocial assessments, and, in some cases, forensic investigations. As such, the designation of abuse used in this study reflects a substantiated status recognized at the national level, rather than a classification based solely on caregiver history or isolated clinical suspicion. The hand fractures in these children were attributed to violence. In the reviewed cases classified as abuse, documentation indicated the father as the alleged perpetrator in each instance.

In reviewing the medical records of children identified as victims of abuse (*n* = 30), additional physical findings beyond the hand fracture were documented in several cases. These included bruises on the upper limbs or torso, superficial abrasions, and soft tissue swelling. However, the presence and documentation of such indicators were inconsistent, and not all cases included detailed descriptions of external injuries. This likely reflects the variability in clinical documentation practices in retrospective records. Nevertheless, the existence of such findings in some cases supports the classification of these injuries as non-accidental (See Table 1).

**Table 1:** Socio-demographic, clinical, and Abuse-related characteristics of the study participants (*N* = 828)

Category	Variable	*n*	%
Demographic Characteristics	Male	459	55.4%
	Female	369	44.6%
	Jewish	489	59.1%
	Arab	339	40.9%
Fracture Cause	Fall	567	68.5%
	Trauma	176	21.3%
	Violence	30	3.6%
	Sports activity	36	4.3%
	Traffic accident	6	0.7%
	Other	13	1.6%
Clinical History	Hospitalizations in past year: 0	766	92.5%
	1	55	6.6%
	2	6	0.7%
	5	1	0.1%
	History of previous fractures: No	778	94.0%
	Yes	50	6.0%
	CT performed: No	815	98.4%
	Yes	13	1.6%
	Surgery performed: No	771	93.1%
	Yes	57	6.9%
	Hospitalized: No	807	97.5%
	Yes	21	2.5%
	Length of stay (days): 0	807	97.5%
	2	11	1.3%
	3	7	0.8%
	4	3	0.4%
Abuse Indicators and Response	Identified as abuse victim: Yes	30	3.6%
	Reported to social worker: No	797	96.3%
	Yes	31	3.7%
	Rehospitalizations: No	795	96.0%
	Yes	33	4.0%
	Death	828	100.0%
	Skeletal survey: No	828	100.0%
	Bone scan: No	828	100.0%
	Source of admission: Home	828	100.0%
	X-ray performed: Yes	828	100.0%

Note: Percentages are based on the total sample size (*N* = 828). Some totals may not sum to 100% due to rounding

Abbreviations: CT = Computed tomography

We compared the characteristics of children with hand fractures who were identified as victims of child abuse, based on official documentation of domestic violence, with those who were not identified as such. Several differences emerged between these two groups. Children of Arab nationality were significantly more likely to be identified as victims of child abuse (93.3%) compared to children of Jewish nationality (6.7%) (χ²=35.33, df = 1, *p* < 0.01). In contrast, no significant differences were observed in gender or age between the two groups, with the average age in both groups being around 3 years (*p* > 0.05).

Children with a history of previous fractures were more likely to be identified as victims of child abuse compared to those with no such history (χ²=418.06, df = 1, *p* < 0.01). In addition, children identified as victims of child abuse had a higher average number of previous fractures compared to those not identified as abuse victims (M = 0.93 vs. M = 0.03; t = 29.02, df = 826, *p* < 0.05). Moreover, children identified as victims of child abuse had a higher average number of hospitalizations during the past year compared to those not identified as victims (M = 1.13 vs. M = 0.05; t=-21.02, df = 826, *p* < 0.01). Finally, children who were rehospitalized following the hand fracture were more likely to be identified as victims of child abuse compared to those who were not rehospitalized (χ²=698.73, df = 1, *p* < 0.01).

As shown in Table 1, the majority of hand fractures occurred due to falls (68.5%), while only a small fraction were attributed to violence (3.6%)- a figure that aligns with the proportion of children officially identified as abuse victims (3.6%). Although prior hospitalizations and history of previous fractures were relatively uncommon across the entire cohort (7.5% and 6.0%, respectively), these factors were markedly more prevalent among children identified as abuse victims. This trend is reflected in the logistic regression results (Table 2), where prior hospitalization (OR = 3.1) and the number of previous fractures (OR = 5.3) were found to be strong predictors of abuse identification. These findings suggest that even rare clinical histories, when present, significantly raise suspicion of abuse and highlight the importance of thorough longitudinal record review in pediatric assessments.

**Table 2:** Logistic regression analysis of predictors for identification of child abuse in children with hand fractures (N   828)

Variable	Odds Ratio (OR)	95% Confidence Interval (CI)	*p*-value
Arab nationality	2.4	1.01–5.93	0.01
History of previous fractures	1.9	1.20–2.99	0.01
Number of previous fractures	5.3	2.03–13.83	0.01
Hospitalizations (past year)	3.1	1.31–7.46	0.01

Model statistics: Nagelkerke R²= 0.21; Model χ²(4) = 29.47, *p* < 0.001

Note: OR = Odds Ratio; CI = Confidence Interval. Significant predictors are bolded

In the logistic regression analysis aimed at determining predictors of identification of child abuse among children with hand fractures, several significant factors emerged (Table 2). Children of Arab nationality were found to be 2.4 times more likely to be identified as victims of child abuse compared to their Jewish counterparts (OR = 2.4; 95% CI: 1.01–5.93; *p* < 0.01). Additionally, children with a history of previous fractures were 1.9 times more likely to be identified as victims of abuse than those without such history (OR = 1.9; 95% CI: 1.20–2.99; *p* < 0.01). Each additional previous fracture further increased the likelihood of abuse identification, with an OR of 5.3 (95% CI: 2.03–13.83; *p* < 0.01). Furthermore, children who had been hospitalized in the year preceding the current injury were 3.1 times more likely to be identified as abuse victims (OR = 3.1; 95% CI: 1.31–7.46; *p* < 0.01).

The overall model was statistically significant (χ²(4) = 29.47, *p* < 0.001), with a Nagelkerke R² of 0.21, indicating that approximately 21% of the variance in identification outcomes was explained by the included predictors.

## Discussion

This study aimed to determine the socio-demographic and medical characteristics associated with identification of child abuse in young children presenting to the ED with hand fractures. Notably, 3.6% of the sample were reported as victims of child abuse, which is similar with findings of Kim and Drake (2018), who noted that 4% of children in the United States are reported to Child Protective Services annually. This similarity could be due to similar healthcare practices in detecting and reporting abuse.

In our study, children of Arab nationality were found to be twice as likely as their Jewish counterparts to be identified as victims of child abuse. This aligns with findings from a previous Israeli study, where a disproportionate 67.5% of child abuse victims were of Arab nationality (Ben Natan et al., 2014). Literature suggests that ethnic minority children may be at a higher risk of being victims of child abuse due to a combination of socioeconomic challenges and cultural norms (Mersky et al., 2021). The societal norms within many traditional communities emphasize respect for authority and the importance of maintaining family honor, which can influence behaviors and attitudes towards discipline. In some cases, this cultural emphasis on authority can exacerbate situations where physical discipline is normalized as an acceptable method of managing children’s behavior (Salami & Alhalal, 2020).

At the same time, it is important to consider the demographic and institutional context in which the study was conducted. The medical center where the data were collected serves a large Arab population, which may contribute to the higher number of identified abuse cases in this group. Additionally, research has highlighted the potential role of implicit or systemic biases in influencing child protection decision-making. These biases—often unintentional—may affect how professionals perceive risk and determine when to initiate reporting procedures. Therefore, the observed disparity should be interpreted with caution, recognizing that it may reflect both actual differences in incidence and differential patterns of identification. Further research across diverse clinical settings is needed to better understand the interaction between ethnicity, healthcare access, and abuse detection.

Moreover, reporting discrepancies may also play a role. Caregivers from different ethnic backgrounds may vary in how and when they seek medical attention, which can influence the types and severity of injuries observed by clinicians. This is particularly relevant in the context of ongoing political conflict and systemic inequalities. Arab families living in peripheral or underserved areas may face logistical, linguistic, or cultural barriers to timely healthcare access, especially for minor injuries. Delayed presentation can lead to more severe clinical findings and may increase the likelihood that abuse is suspected (Palusci & Botash, 2021; Cénat et al., 2021).

Beyond cultural and reporting differences, broader structural factors may contribute to the disparity observed between ethnic groups. Arab communities in Israel, particularly in peripheral regions, often experience systemic disadvantages, including limited access to healthcare services, lower average socioeconomic status, and underfunded infrastructure. These inequities can result in delayed care-seeking, fewer routine health encounters, and reduced trust in healthcare institutions- factors that may influence both the incidence and recognition of abuse (Ben Natan et al., 2014; Cénat et al., 2021). Furthermore, regional tensions and political marginalization may exacerbate institutional biases or contribute to inconsistencies in child protection responses Cénat et al., 2021; Palusci & Botash, 2021. A more equitable allocation of healthcare and social resources, alongside culturally sensitive training for providers, is essential for mitigating these disparities and ensuring fair identification and protection across populations (Ben Natan et al., 2014).

The higher likelihood of children of Arab nationality being identified as victims of child abuse may reflect greater visibility and, consequently, more effective identification of abuse cases within this community. However, this disparity could also stem from biases. Racial biases have been shown to contribute to the overrepresentation of certain groups in the child welfare system, increasing their chances of being reported, investigated, and placed in care (Cénat et al., 2021).

In this study, all documented cases of abuse were attributed to paternal figures. While this observation may reflect reporting or contextual factors and cannot be generalized, prior literature has noted the role of paternal figures in adverse family dynamics. For example, Adhia and Jeong (2019) found that fathers’ perpetration of intimate partner violence was associated with reduced positive parenting practices during early childhood. These findings suggest that exposure to paternal aggression, even if not directed toward the child, may signal broader risks within the family environment and highlight the need for vigilance in such contexts. In many traditional cultures, gender roles and societal expectations often position the father as the dominant figure within the family structure (Novianti et al., 2023). Such entrenched power imbalances may, under certain conditions—such as elevated stress, substance abuse, or mental health challenges—manifest in abusive behavior (Hendaus et al., 2020).

Our study found no significant association between the age or gender of children with hand fractures and the likelihood of being identified as victims of child abuse. This finding is consistent with that of Ben Natan et al. (2014), who also reported no significant gender-based differences in abuse identification. However, this lack of association should be interpreted with caution. The relatively narrow age range (0–5 years) and limited sample size may reduce variability and statistical power, potentially masking meaningful differences. Moreover, in young children, especially infants and toddlers, developmental limitations may constrain opportunities for both accidental injuries and verbal disclosure, making age-based differences in abuse detection harder to observe.

Comparisons with broader studies reveal mixed findings. Some studies have reported higher abuse risk among infants and male children (Walz et al., 2024), while others found no consistent association (Ben Natan et al., 2014). These discrepancies highlight the importance of considering contextual, cultural, and reporting factors when interpreting demographic patterns in abuse identification.

In the present study, children with a history of previous fractures were more likely to be identified as a victim of child abuse compared to children without such a history. Moreover, each additional previous fracture significantly increased the likelihood of the child being identified as a victim of abuse. Previous traumatic injuries should trigger clinical suspicion of child abuse (Milner et al., 2022). These findings suggest that healthcare providers are effectively using a child’s medical history in their practices for identifying potential abuse.

Similarly, in our study, children who had been hospitalized in the year preceding their current injury were more likely to be identified as victims of child abuse. This aligns with recommendations for healthcare providers to be vigilant in suspecting abuse when a child exhibits a pattern of frequent hospital visits, particularly if these visits are related to injuries or conditions commonly associated with maltreatment (Liu et al., 2022).

Notably, in our study, all cases of hand fractures among children identified as victims of child abuse were attributed to violence. This suggests that healthcare providers may be more adept at recognizing abuse when the injury clearly aligns with violent circumstances, thereby influencing their diagnostic and reporting practices. It has been previously suggested that healthcare providers are more attuned to identifying abuse in cases where injury patterns align with known markers of abusive behavior (Quiroz et al., 2021). However, this raises concerns about the thoroughness of assessments in situations where evidence of abuse is less apparent, potentially allowing some instances to go unnoticed.

While this heightened diagnostic accuracy may reflect a more precise identification of obvious abuse, it may also contribute to a diagnostic bias. Providers may be less likely to consider non-violent causes of injury as potential indicators of abuse, which could lead to under-recognition in cases involving unclear injury mechanisms or primary concerns such as emotional abuse or neglect. Addressing this issue requires further research to understand the factors influencing providers’ decision-making processes.

Importantly, the experience of secondary trauma among mandated reporters should not be viewed solely through an individual psychological lens. Inadequate institutional recognition, lack of structured debriefing protocols, and limited training in trauma-informed care represent systemic shortcomings that may amplify the emotional toll on professionals involved in abuse detection. When institutions fail to provide clear guidelines, multidisciplinary collaboration, or psychological support mechanisms, professionals may feel isolated or unsupported- potentially delaying or avoiding mandatory reporting (Manan et al., 2022; Palusci & Botash, 2021). These systemic gaps not only reflect operational deficiencies but also raise ethical concerns, as they may hinder child protection and perpetuate under-recognition of abuse. Institutional accountability is therefore essential for ensuring both effective abuse identification and the emotional resilience of those tasked with this responsibility (Cénat et al., 2021; Ben Natan et al., 2014).

Although certain variables in our analysis did not reach statistical significance—such as age, gender, and the specific cause of injury- they may still carry clinical relevance. Previous literature has reported that boys and infants are often overrepresented in physical abuse cases (Walz et al., 2024), while other studies found no consistent associations (Ben Natan et al., 2014). Similarly, although most fractures in our cohort were attributed to falls, providers should be attentive to injury patterns or explanations that are inconsistent or vague, even when classified under common mechanisms. These factors, although not predictive in isolation, may contribute meaningfully to clinical suspicion when viewed within the broader context of injury history, caregiver behavior, and psychosocial background (Milner et al., 2022). Therefore, their potential contribution to risk assessment should not be disregarded in practice.

To mitigate this bias, comprehensive training programs for healthcare providers should be implemented, focusing not only on identifying physical signs of abuse but also on recognizing the less overt markers of neglect and emotional abuse. Additionally, standardizing assessment protocols and fostering closer collaboration with social services could ensure that all forms of child abuse are thoroughly evaluated, regardless of whether violence is the apparent cause of the injury.

While this study focused on identification patterns within a clinical setting, it is important to acknowledge that mandated reporters extend beyond healthcare professionals. Educators, child welfare workers, and religious figures are also often responsible for reporting suspected abuse, and they may experience secondary trauma differently. These non-clinical professionals often operate within institutions that provide limited access to psychological support or structured debriefing practices. Future research should explore the unique challenges faced by these professionals and how institutional context shapes their ability to detect and respond to child abuse effectively.

## Limitations

Despite the insights gained from this study, several limitations should be acknowledged. First, the retrospective nature of the study relies heavily on accurate and complete medical records, which may be subject to documentation errors or missing data. Variability in record-keeping practices across different healthcare providers could also affect the consistency and reliability of the data. Second, the study focused exclusively on children presenting with hand fractures to a single pediatric emergency department in north-central Israel. This limits the generalizability of the findings to other settings with different demographic compositions, healthcare practices, or cultural contexts. Third, the study did not explore the perspectives of healthcare providers regarding their practices in identifying and reporting child abuse cases. Understanding the challenges and decision-making processes from their viewpoint could provide additional insights into the factors influencing the identification of abuse. Fourth, while the study identified significant associations between certain socio-demographic and medical factors and the identification of child abuse, causality cannot be inferred due to the observational nature of the study design. Other unmeasured variables or confounding factors may also influence the outcomes.

The higher rate of abuse identification among Arab children in this study should be interpreted cautiously. Since the study was conducted at a medical center that serves a predominantly Arab population, this may have influenced the proportion of identified cases. Moreover, the potential presence of implicit or systemic biases in abuse reporting and identification cannot be ruled out. Multicenter studies across more diverse settings are needed to validate and contextualize these findings.

Another limitation of this study is the absence of data regarding the demographic or professional characteristics of the medical providers involved in the evaluation and reporting process. Factors such as provider training, years of experience, cultural background, or personal biases could influence abuse identification and substantiation rates. Future research should consider the role of provider characteristics and their potential impact on decision-making in suspected abuse cases.

An additional limitation is the lack of detailed classification of hand fracture types in the dataset. Fracture patterns (e.g., transverse, oblique, spiral, comminuted) were not consistently documented in the medical records and were therefore not analyzed. This restricts the ability to explore whether certain fracture types are more indicative of non-accidental trauma. Incorporating detailed radiographic data — such as fracture configuration and mechanism — could enhance early recognition of high-risk injury patterns and serve as a valuable training tool for clinicians in emergency settings. Future studies should incorporate radiographic classifications to determine whether specific fracture patterns are associated with abuse, which could improve diagnostic accuracy and clinical decision-making.

Finally, another limitation of the study is the absence of skeletal surveys in the evaluated cases, despite this being a key imaging modality recommended in the assessment of suspected physical abuse in young children. The lack of skeletal surveys may reflect local institutional practices, limited awareness or inconsistent adherence to international guidelines among healthcare providers, particularly in cases where the initial clinical suspicion of abuse was low. As such, it is possible that some occult injuries went undetected, which may have led to under-identification of abuse. This highlights the need for increased provider training and standardized imaging protocols in suspected abuse cases to improve detection and reporting.

## Conclusion

This study investigated the socio-demographic and medical characteristics associated with the identification of child abuse in young children presenting with hand fractures. The findings revealed that nationality, a history of previous fractures, and prior hospitalizations significantly increased the likelihood of these children being identified as victims of child abuse. The study highlighted that fathers were the primary perpetrators in identified abuse cases. These results emphasize the importance of thorough assessment by healthcare providers, especially when dealing with recurrent injuries and hospitalizations. While the study provides valuable insights, Further research is needed to validate these findings in other cultural and clinical contexts and to explore how provider training, bias, and systemic variables shape the identification and reporting of child abuse.

## Data Availability

Data is available on request.
